# Fruit and Leaf Response to Different Source–Sink Ratios in Apple, at the Scale of the Fruit-Bearing Branch

**DOI:** 10.3389/fpls.2019.01039

**Published:** 2019-08-28

**Authors:** Emna Baïram, Christian leMorvan, Mickaël Delaire, Gerhard Buck-Sorlin

**Affiliations:** ^1^INRA, Unité PSH, Domaine St Paul, Agroparc, Avignon, France; ^2^IRHS, INRA, Agrocampus-Ouest, Université d’Angers, Quasav, Beaucouzé, France

**Keywords:** *Malus x domestica* Borkh, apple, branch, source–sink ratio, photosynthesis, defoliation, functional-structural plant modeling, fruit growth

## Abstract

Apple fruit growth is the result of several factors: inherent demand (relative sink strength) of the fruit (defined by the demands for cell division and expansion growth, etc.), carbon assimilation by the source leaves (source strength), and the resulting allocation to the organ in question. It is thus a complex process involving source–sink interactions. In the present study, we designed an experimental system in which parts of fruit-bearing branches of two apple cultivars (“Fuji” and “Ariane”) were isolated from the rest of the tree by girdling and then subjected to specific pruning and fruit removal treatments to create a wide range of global (branch-level) source–sink ratios. We monitored not only fruit kinetics but also photosynthesis as a response to light in leaves of the three different shoot types (i.e., the rosette, the bourse, and the vegetative shoots) to 1) study the impact of source–sink distance on carbon partitioning between fruits within the same branch and 2) to investigate the impact of source/sink ratio on fruit growth and leaf photosynthetic activity. Our results indicate 1) no significant differences among lateral fruits belonging to different ranks, and this independent of source availability; 2) that a modification of the source/sink ratio seems to be compensated by an alteration of the photosynthetic rate of leaves, with stronger and weaker values obtained for lower and higher ratios, respectively. Moreover, our results seem to suggest that two growing sinks together will upregulate photosynthesis rate more strongly than one growing sink does on its own, and this with the same leaf area per fruit. These results are discussed, and some hypotheses are put forward to explain them.

## Introduction

One of the most important parameters for fruit quality is its size at harvest. From the sites of its production or remobilization (source organs such as leaves or reserve tissues), carbon is transported to the sink organs (more specifically the fruits) where it accumulates and is involved in the growth process. A qualitative appreciation and ultimately, quantification of carbon transport and distribution in the frame of variable source–sink relations during fruit development, should help to better understand or even predict, fruit size, and quality at harvest. Carbon transport within the fruit tree is, thus, a complex process: its study is requiring the consideration of both structural components linked to tree architecture, and functional ones linked to source–sink relations and assimilate production and transport. In apple, this complexity is increased by the diversity of the vegetative shoot types (long, short, proleptic, and sylleptic: Costes, 2003), the age structure of the branch wood carrying the sink organs, and the composition of the fruit-bearing units. In any case, the appreciation of these phenomena constitutes a major problem to be resolved, ultimately to have a better insight into the basic processes underlying fruit growth and quality.

**Figure 1:**
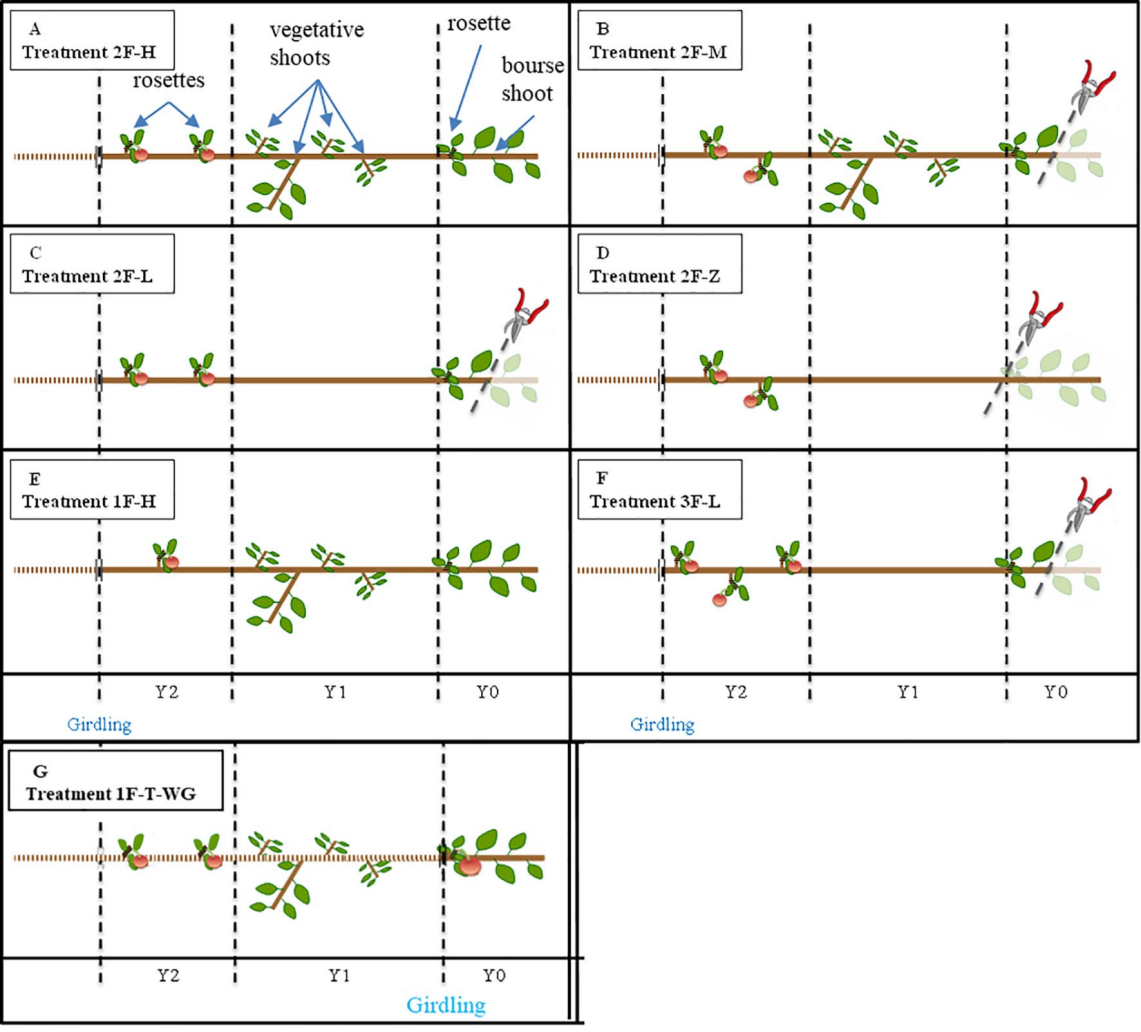
Schematic representation of the treatments applied to experimental units. Applied pruning treatments are: 2 axillary fruits and “high” leaf area on the branch (2F-H, Panel **A**), 2 axillary fruits and ‘medium’ leaf area on the branch (2F-M, Panel **B**), 2 axillary fruits and “low” leaf area on the branch (2F-L, Panel **C**), 2 axillary fruits and almost ‘zero’ leaf area on the branch (2F-Z, Panel **D**), 1 axillary fruit and “high” leaf area on the branch (1F-H, Panel **E**), 3 axillary fruits and “low” leaf area on the branch (3F-L, Panel **F**), control with girdling below Y0, 1 terminal fruit and unreduced leaf area (1F-T-WG, Panel **G**). In (“Fuji,” “Ariane”), N = (9,8); (7,8); (8,8); (7,6); (9,0); (8,0) for “2F-H,” “2F-M,” “2F-L,” “2F-Z,” “1F-H,” “3F-L,” respectively. Y2, Y1 and Y0 refer to the first order shoot of 2013, 2014, and 2015, respectively. A girdling was applied at the base of Y2 (except for 1F-T-WG, see above), and all shoots except the rosettes supporting the fruits considered in the experiment were removed from Y2. In “2F-H,” “2F-M,” “2F-L,” and “2F-Z,” a thinning to two fruit-bearing spurs was made, in “1F-H” and “3F-L,” a thinning to one and three fruit-bearing spurs, respectively was applied. Bourse shoots were removed from the experimental spurs and a thinning to one fruit per spur was made in Y2. All fruit-bearing spurs were removed from Y1, and in “2F-L,” “2F-Z,” and “3F-L,” all shoots were removed from Y1. In “2F-M,” “2F-L,” and “3F-L,” the bourse shoot(s) of Y0 was pruned at the level of the first half of its internodes. All fruits were removed from Y0 (except for 1F-T-WG).

Dedicated ecophysiological experiments provide some evidence that the position of an apple in the tree crown and more specifically, on the fruit-bearing branch has repercussions on its growth and quality, e.g., fruit weight (Reyes et al., 2016), fruit color (Robinson and Lakso, 1988), soluble solids content (Campbell and Marini, 1992), or general fruit quality traits (Wagenmakers and Callesen, 1995; Wagenmakers and Callesen, 1988). We define the fruit-bearing branch as a first-order branch (or part of it), which is composed of three types of shoots: rosette (bourse), bourse shoot, and vegetative shoot (**Figure 1**). Effectively, depending on its position, the fruit is surrounded by a specific microclimate (Chelle, 2005), but also exhibits a unique topological and geometrical distance to the nearest leaf (source) as well as to competing sinks that could be decisive for fruit growth and quality. Fruit growth is determined by the availability of assimilates (source) and its ability to attract assimilates, which in turn depends on its sink strength. It is well known that apple fruit sink strength is determined during early fruit development (i.e., cell division) and depends in part on sink competition at the spur level (Goffinet et al., 1995; Wünsche and Lakso, 2000a; Wünsche and Lakso, 2000b). In later development (i.e., during cell expansion), fruit growth likely depends on the ratio between sources and sinks (i.e., source availability for each sink) and distance between sources and sinks, as observed in several fruit species such as peach (Bruchou and Génard, 1999). However, in apple, there is some evidence that the distance between source and sink is not very decisive for carbon distribution (Hansen, 1969). Furthermore, under conditions of a high source–sink ratio, leaf photosynthetic activity in apple leaves could possibly be reduced due to lower carbon demand, which in turn could lead to a carbon budget comparable to one obtained under a moderate source–sink ratio (Palmer et al., 1997; Pallas et al., 2018). This result points out the necessity to take into account the real carbon production in apple, to determine the amount of carbon available for fruit growth and quality, or at least to know the impact a given source/sink ratio has on photosynthetic activity to obtain an approximate idea of carbon availability. However, as stated before, microclimate must also be taken into account to improve the accuracy of prediction of photosynthesis, as each individual leaf, due to its position, size, and orientation in space, might experience a different light regime, and therefore, photosynthetic rate dynamics, during the day and during an entire season. The reconstruction of branch architecture plus microclimate necessitates a 3D modeling approach: Functional-Structural Plant Modeling (FSPM, Vos et al., 2010; Buck-Sorlin, 2013) is a suitable modeling method that considers plant architecture in 3D, as well as basic physiological and biophysical processes (light interception, photosynthesis, growth, respiration, and so on) at the organ scale. The use of such models can be challenging with respect to the rather tedious data acquisition, which is a real bottleneck. Recently published work by Baïram et al. (2017) proved that accurately modeling leaf area could be a very valuable first step for the reconstruction of branch architecture and leaf area as input to a static FSPM.

Given this global context, the objectives of this work, which was conducted at the scale of the fruit-bearing branch, were twofold: 1) to study the impact of source–sink distance on carbon partitioning to confirm or not the results obtained by Hansen (1969); 2) to investigate the impact of source/sink ratio on fruit growth and leaf photosynthetic activity. The present work was conducted in the frame of the doctoral thesis of the first author (Baïram, 2017), with the overall aim to identify the role of different source and sink organs for the buildup of fruit quality, at the spur and branch level.

## Materials and Methods

### Plant Material

The experiments were carried out in 2015 on apple trees planted in 2008, at the INRA experimental unit in Beaucouzé, France (47° 28′ N, 0° 37′ W). Two cultivars were selected for this experiment: “Fuji” and “Ariane.” “Ariane” is a modern cultivar that was developed by INRA Angers, by crossing a hybrid of “Florina” x “Prima” with pollen from “Golden Delicious,” and released in the year 2000, as a scab-resistant variety. The “Fuji” cultivar is a hybrid developed by growers at Tohoku Research Station in Fujisaki, Aomori, Japan, in the late 1930s and has been commercialized since 1962. The origin of this cultivar is two American apple varieties: “Red Delicious” and “old Virginia Ralls Genet.” The main aim of these experiments was to study the effect of source/sink balance on fruit-bearing sub-branches (FBB) of apple trees. Therefore, 47 FBB of “Fuji” and 30 of “Ariane” were selected. In this study, we refer to “sub-branch” as the part of a first-order fruit-bearing branch comprising three annual extensions. A first-order branch corresponds to a sylleptic branch formed directly on the main stem during the juvenile phase of the tree. It consists of long and short vegetative shoots, as well as bourses and bourse shoots. Each sub-branch is composed of a single-order axis composed of one 2-year-old shoot (Y2), one 1-year-old shoot (Y1), and one current Y0 unit corresponding to a spur (composed of rosette leaves, a bourse, a bourse shoot, and fruits) in terminal position and several ramifications in axillary position on Y2 and Y1 corresponding to spurs or vegetative shoots. One week before the onset of the experiment, all sub-branches were thinned to one fruit—the king fruit—per spur. Afterward, several defoliation and/or fruit-thinning treatments were applied to test the impact of source/sink ratio on fruit growth and photosynthetic activity.

### Experimental Design

The experimental design was based on a completely randomized selection of sub-branches (**Figure 1**). Considering that apple leaves are photoassimilate sinks at the beginning of their development as shown by Grappadelli et al. (1994), experiments were conducted once all the leaves were fully developed to be sure that leaves exclusively exerted the function of a source. Treatments were applied on June 22, 2015, 67 and 63 days after full bloom (i.e., DAFB) for “Ariane” and “Fuji,” respectively, after all vegetative or bourse shoots were completely developed. A girdling was applied at the base of Y2, and all fruits from Y0, all fruiting spurs born in axillary position on Y1, and all vegetative or bourse shoots and non-fruiting spurs born on Y2 were removed. Therefore, the experimental design was such that all fruits (sinks) were located on Y2 and vegetative shoots and bourse shoots on Y1 and Y0 only. The girdling treatment was applied to have a closed system with respect to the carbon budget for which we could compute the carbon balance, knowing that under this treatment, no carbon can be exported to the rest of the tree.

Afterward, four different leaf/fruit ratio treatments were applied on both “Fuji” and “Ariane” with between six and nine replicates for each treatment. For each treatment, two spurs bearing one fruit on Y2 were kept, and leaf/fruit ratios were modulated by shoot pruning applied to Y1 and Y0 to have i) high, ii) medium, iii) low, and iv) very low source availability: i) no shoot pruning (treatment “2F_H,” **Figure 1A**); ii) removal of half of the bourse shoot on Y0 (treatment “2F_M,” **Figure 1B**); iii) removal of half of the bourse shoot on Y0 and of all shoots on Y1 (treatment “2F_L,” **Figure 1C**); iv) removal of all shoots and non-fruiting spurs on Y1 and Y0 (treatment “2F_Z,” **Figure 1D**).

Two supplementary treatments were applied on “Fuji” to test extreme leaf/fruit ratios by v) keeping only one fruit-bearing spur on Y2 while applying no shoot pruning (treatment “1F_H,” **Figure 1E**) and vi) keeping three fruit-bearing spurs on Y2 while pruning half of the bourse shoot on Y0 and all shoots on Y1 (treatment “3F_L,” **Figure 1F**).

For all treatments, rosette leaves were kept on fruiting spurs (on Y2) to limit fruit drop between the beginning of the experiment and harvest.

Experiments carried out at the sub-branch scale were completed by an experiment conducted at the spur scale using twenty intact spurs bearing one fruit in terminal position (Y0) and isolated from the rest of the branch by girdling (treatment “1F-T_WG,” **Figure 1G**). This experiment, which was started on June 30, 2015, was designed to check if structures comprising a fruit with nearby sources on the same spur would behave differently from the structures considered above, and to compare the characteristics of terminal fruit to fruits born at an axillary position on the branch.

### Fruit Growth Survey

At the beginning of the experiments and until harvest, all fruit diameters were surveyed once every 1 to 2 weeks. Fruit growth was expressed as developmental time from the date of full bloom to take into account the different dates of anthesis for each cultivar. Developmental time was first measured using growing degree hours [GDH, with a base temperature *T_b_* = 7°C (Anderson and Richardson, 1982)] calculated (equation 1) using hourly air temperatures (°C) obtained from the weather station of Beaucouzé (47° 28′ N, 0° 37′ W, 50 m a.s.l.) and accessed from the INRA Climatik platform, https://intranet.inra.fr/climatik_v2:

(1) $$GDH_{i}=\sum_{h=1}^{24}\left(HT_{h}-T_{b}\right)$$

where *HT_h_* is replaced by *T_b_* if *HT_h_* < *T_b_*; *HT_h_* is the hourly air temperature at hour *h*; and *GDH_i_* are the GDHs on day *i*. In this study, the thermal time variable expressed in GDD was normalized to cumulated thermal time (GDD_cum_) after full bloom (equation 2).

(2) $$GDD_{cum}=\sum_{i=FB}^{D}\frac{GDH_{i}}{24}$$

where *FB* is the day of full bloom, and *D* is the number of days since *FB*. Full bloom occurred on April 16, 2015, in “Ariane” and on April 20, 2015, in “Fuji.”

At harvest, all fruits were collected together with their bearing bourse and both organ diameters, heights, volumes, fresh and dry weights measured. Fruit circumferences were also measured. All harvested fruits were cut, and then dried during 6 days at 60°C in a ventilated oven (type HORO 900 V/RS, Dr. Hofmann GmbH, Stuttgart, Germany).

As a control, the diameters of each fruit born on Y2 for each sub-branch were compared at the beginning of experiments to be sure that fruits were equivalent in size to exclude that a difference in fruit size at harvest could be caused by an initial difference.

### Leaf Area Estimation

For each treatment, total leaf area (TLA) of vegetative shoots (VS), bourse shoots (BS) and rosettes (RO) was estimated using the models established by Baïram et al. (2017): these models consider the type of shoot (*j* = RO, BS, VS) and the genotype (*G*) and use two variables as input: the length of the biggest leaf (*L*_max_) of a shoot and its number of leaves (*nl*). Except for bourse shoots on Y0, which were half pruned (i.e., “2F-M,” “2F-L,” “3F-L” treatments), TLA was calculated using equation 3 with parameters ß and *k*, depending on shoot type and genotype (**Table 1**). For non-fruiting spurs kept on Y1 and Y0, TLA was calculated as the sum of TLA obtained for RO and BS.

(3) $$TLA=\beta_{j,G}\cdot nl\cdot \frac{\pi }{4}\cdot k_{j,G}\cdot L_{max}^{2}$$

**Table 1:** *ß* and *k* parameters used in computing TLA for each shoot based on shoot type and genotype.

		G			
		‘Fuji’		‘Ariane’	
		*β*	*k*	*β*	*k*
*j*	RO	0.69	0.74	0.69	0.75
	BS	0.67	0.64	0.67	0.60
	VS	0.48	0.56	0.48	0.56

TLA, total leaf area; RO, rosette; BS, bourse shoot; VS, vegetative shoot (Baïram et al., 2017).

In equation 3, *nl* [-] is the number of leaves on the shoot, *L_max_* [cm] is the length of the biggest leaf on the branch, *k_j,G_* [-] and *β_j,G_* [-] are both shoot type (j)- and genotype (G)-dependent dimensionless parameters of the model provided by Baïram et al., 2017.

Estimation of TLA of the bourse shoots half pruned in terminal position (i.e., Y0) was computed using equation 4 where α*_BS,G_* was equal to 0.99 and 1.20 in “Fuji” and “Ariane,” respectively, and values for parameter *s_BS,G_* were a function of leaf number as described by Baïram et al., 2017. α*_BS,G_* [-] is also a shoot type (j)- and genotype (G)-dependent parameter of the model described by equation 4 and *R* is the rank of the leaf on the shoot. *s* [-] defines the slope of the curve of the Lorentz function used in the model of Baïram et al. (2017).

(4) $$\int_{\frac{-2}{nl}}^{\frac{\frac{nl}{2}-2}{nl}}\alpha_{BS,G}\frac{\frac{\pi }{4}\times k_{BS,G}\times L_{max}^{2}}{1+\frac{{\left(R-2\right)}^{2}}{nl^{2}\times s_{BS,G}^{2}}}.dR$$

Comparisons of estimated TLA among treatments were made for “Fuji” and “Ariane,” respectively, to establish if treatments were in fact different among different types of pruning. Leaf area prediction using our model was very accurate, with coefficients of determination of R^2^ = 0.95 and 0.89 for bourse shoots of “Ariane” and “Fuji,” respectively (for rosettes and vegetative shoots, R^2^ = 0.97 and 0.83, respectively, in both genotypes). For details, see Baïram et al., 2017.

### Measurement of Instantaneous Net Photosynthesis Rate

Between July 15, 2015, and July 31, 2015, CO_2_ exchange rate measurements were made on: i) three leaves per shoot of three bourse shoots subjected to treatments “1F_H” (noted “BS_1F_H”); ii) two or three leaves per shoot of three bourse shoots subjected to treatments “3F_L” (noted “BS_3F_L”); iii) two or three leaves per shoot of two bourse shoots for terminal spurs bearing one fruit and with basal girdling (noted “BS_T_WG”); iv) three or four leaves per shoot of two bourse shoots for terminal spurs bearing one fruit and without basal girdling (noted “BS_T_NG”); v) one rosette leaf of a fruit-bearing spur for treatment “2F_Z” (noted “RO_2F_Z”); vi) one or three rosette leaves of three terminal spurs bearing one fruit and with basal girdling (noted “RO_T_WG”); vii) three rosette leaves of two terminal spurs bearing one fruit and without basal girdling (noted “RO_T_NG”); and viii) one leaf per shoot of nine vegetative shoots for treatments “1F_H” (noted “VS_1F_H”), yielding a total of 51 light-response curves. Net CO_2_ assimilation rate was measured as a response to a range of 11 to 13 levels of irradiance going from 2,000 down to 0 μmol·m^2^·s^−1^ of photosynthetically active radiation (PAR) using a portable LI-6400XT infrared gas analyzer photosynthesis system (Li-Cor, Lincoln, NE, USA) equipped with a standard chamber that allowed the clamping of a 9 cm² leaf surface. Measurements were made only on “Fuji” leaves, between 8:00 am and 12 am, with the initial measurement (at 2000 µmol) taking between 3 and 15 min, thereby allowing the leaf to acclimatize to the saturating PAR. The following nine measurements (at 1,500, 1,000, 700, 500, 250, 100, 50, 25, and 10 µmol PAR) were taken at a frequency of about one every 30 s, while the measurement at 0 µmol PAR lasted between 1 and 5 min. During all measurements, leaf temperature was kept constant at 20°C, CO_2_ concentration at 400 ppm, and RH at 59 ± 6%. Measurements were logged continuously using the monitoring software of the device. Photosynthetic light response curves were then parameterized for each leaf using the model described in equation 5 (see below).

### Data Analysis

#### Modeling of Photosynthesis

We modeled the instantaneous net photosynthesis rate [µmol CO_2_ m^-2^ leaf surface s^-1^] for each simulated leaf using the model proposed by Marshall and Biscoe (1980), which is a non-rectangular hyperbola (equation 5).

(5) $$P_{n}=\frac{(P_{\max}+\alpha PAR-\theta R_{d})\pm \sqrt{{\left(P_{\max}+\alpha PAR-\theta R_{d}\right)}^{2}-4\theta (\alpha PAR(P_{\max}-(1-\theta )R_{d})-R_{d}P_{\max})}}{2\theta }$$

where *P_n_* is the photosynthesis rate (µmol CO_2_.m^-2^ leaf surface.s^-1^) modeled as a function of PAR (µmol photons·m^−2^ leaf surface·s^−1^); *P*_max_ (µmol CO_2_·m^−2^ leaf surface·s^−1^) is the PAR saturated maximum photosynthesis rate; α (µmol CO_2_. µmol photons^-1^) is the photochemical efficiency at low PAR intensities, θ (꼨−) is a scaling parameter describing the ratio of physical (boundary layer–stomatal–mesophyll) to total resistance (physical plus chemical resistance, the latter characterizing the biochemical reaction) to diffusion of CO_2_; and *R_d_* is the dark respiration rate (µmol CO_2_·m^−2^ leaf surface·s^−1^). Normally, the maximum photosynthesis rate *P*_max_ is a function of leaf age and air temperature. However, as we measured photosynthesis rates only at one temperature and on recently mature leaves (age, 20–30 days) we assumed, for simplicity’s sake, that *P*_max_ did not vary over most of the temperature and leaf age range observed in the field.

The *nls* function in R was used to compute three of the four model parameters to make the model fit the data. Initial parameters for the nls function were fixed to *P*_max_ = 12, α = 0.04 and θ = 0.8 with ranges of (5, 25), (0.01, 0.1), and (0.2, 1) for each parameter, respectively. *R_d_* was set as the absolute value of the measured response under conditions of darkness. Impact of treatments on photosynthetic light response was studied by testing the four parameters fixed for each leaf light response using analysis of variance (ANOVA) or Kruskal–Wallis, depending on whether the distribution of residuals was following a normal distribution or not. In general, we used SNK as a *post hoc* test after ANOVA, and the Kruskal test after Kruskal–Wallis. SNK is derived from Tukey, but it is less conservative (finds more differences). Tukey controls the error for all comparisons, where SNK only controls for comparisons under consideration. Kruskal makes the multiple comparisons with Kruskal–Wallis. This *post hoc* test is using the criterion Fisher’s least significant difference. The adjustment methods include the Bonferroni correction and others. The default level of alpha was 0.05 in both tests.

#### Modeling of Fruit Growth Kinetics

A growth curve for each fruit that did not drop before harvest was fitted using the negative exponential model (equation 6). This model was first described by von Bertalanffy (1938) and used by Zadravec et al. (2014). Concerning fruit growth dynamics, the von Bertalanffy equation (VBE, cited in Zadravec et al., 2014 and Tijskens et al., 2015) instead of the expolinear equation proposed by Lakso et al. (1995) was used. Effectively, VBE describes the later stages of fruit growth better than the expolinear equation does, while the latter is more suitable for the early stages of fruit growth where cell division is still ongoing, and during the transition from exponential to linear growth.

(6) $$D(t)=D_{max}(1-e^{\left(-e^{s}(t-x0)\right)})$$

where *D(t)* is fruit diameter at time *t* [days after full bloom]; *D*_max_ is the asymptote of the curve (final fruit size, [mm]); *s* describes the slope of the curve and is related to growth rate; and *x0* [days after full bloom] is the intercept of the curve with the x-axis. The *nls* function in R was used to compute two of the three model parameters to make the model fit the data. Initial parameters for the *nls* function were set to *s* = -7 and *x_0_* = -700. *D_max_* was set as the highest measured value of fruit diameter. *D_max_* was generally measured at harvest when diameter was considered maximal; however, some fruits shrank at the onset of senescence: for those, *D_max_* corresponded to their diameter before they started decreasing in size. Treatment effect on fruit growth was evaluated using tests on the impact of treatments on parameters *D*_max_ and *s* for their biological meanings. These tests were conducted using ANOVA when the distribution of residuals was following a normal distribution or Kruskal–Wallis when this distribution was not normal.

#### Competition Between Sinks for Sources

Competition between fruit was analyzed by testing the impact of vicinity of the fruit to sources on five parameters: i) *D_max_*; ii) *s*; iii) total fresh weights (FW); iv) total dry weights (DW) of the fruit at harvest; v) and dry matter content (DMC) of the fruit as the ratio of its dry and fresh weight at harvest. As we assume that differences in the former parameters may also result from the amount of leaf area available per fruit, the tests were conducted using a two-way ANOVA with both vicinity and total available leaf area per fruit (*ALA*, calculated as TLA on the branch divided by the number of fruit) as factors.

#### Studying the Interaction Between Sources and Sinks

The five parameters describing fruit response to treatments were selected, and their distribution as a function of the leaf area available per fruit (ALA) was graphically represented. Then, tests were made on axillary fruit to check if ALA had an impact on the five parameters cited above for each group of i) “Fuji,” one axillary fruit/branch; ii) “Fuji,” two axillary fruit per branch; iii) “Fuji,” three axillary fruit per branch; and iv) “Ariane,” two axillary fruit per branch.

## Results

### Characterization of Leaf Area

**Figure 2:**
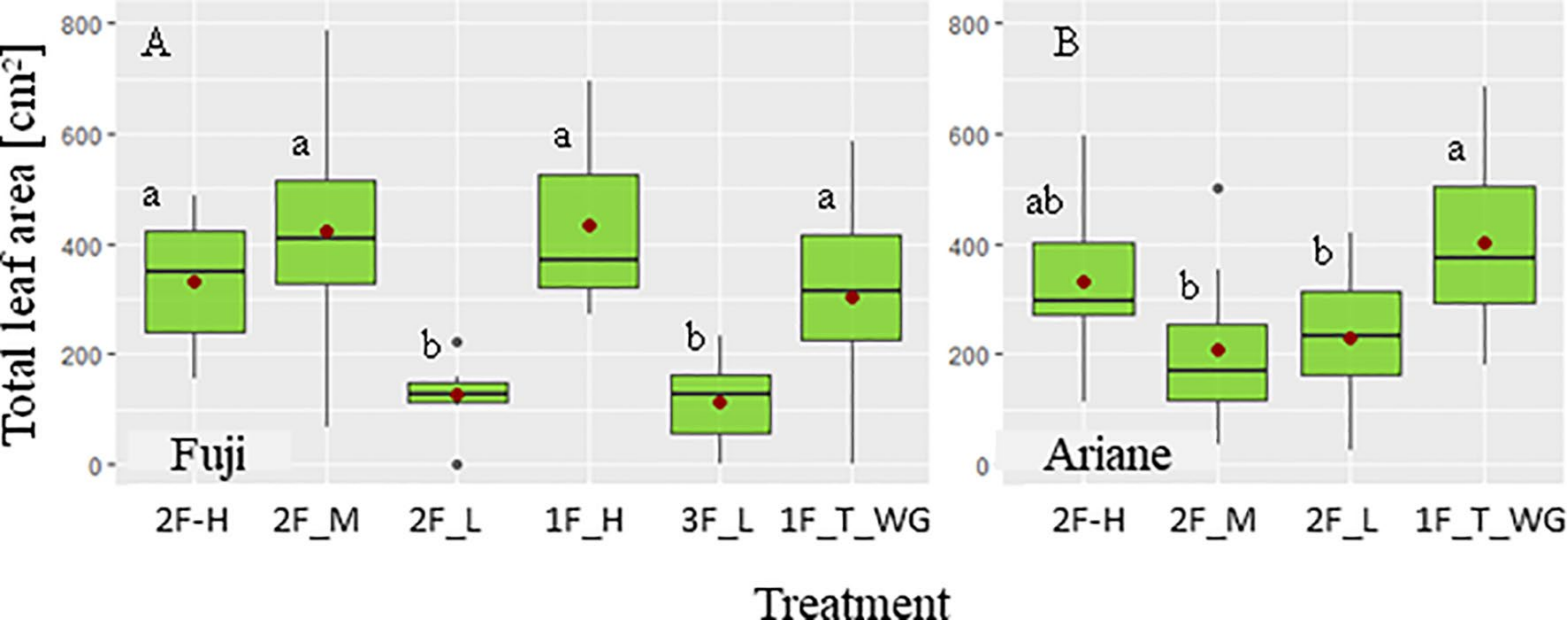
Boxplots of total leaf area (TLA, cm^−2^) as a function of different pruning and defruiting treatments on ‘Fuji’ **(A)** and ‘Ariane’ **(B)** branches: one and two axillary fruits and high TLA (“1F_H” and “2F_H”), two axillary fruits with medium (“2F_M”) and low TLA (“2F_L”), three axillary fruits with low TLA (“3F_L”), one fruit on the terminal spur (“1F_T_WG”). (•) mean value of TLA for each treatment. Treatments with the same letter were considered to be non-significantly different according to one-way ANOVA and/or Kruskal–Wallis results (*P* < 0.05).

Only statistically significant (*P* < 0.05) results are presented in the following if not stated otherwise. Globally, TLA of the branch differed among some treatments in both “Fuji” and “Ariane” (**Figure 2**). In “Fuji,” TLA under “low” treatments (“2F_L” and “3F_L”) was lower than TLA obtained under “high” (“1F_H” and “2F_H”) and “medium” (“2F_M”) treatments, which in turn were not otherwise different amongst each other. Moreover, TLA of “high” and “medium” treatments did not differ from TLA of terminal spurs on Y0 under the “1F_T_WG” treatment. In “Ariane,” no difference in TLA was observed among treatments except for “1F_T_WG,” which exhibited a higher value compared to “medium” and “low” treatments with two fruit per branch. These differences between cultivars could be explained by different leaf area distributions among cultivars with more leaf area on Y0 shoots in “Ariane” compared with “Fuji” (data not shown). In conclusion, these results seemed to show that pruning did not induce clear differences in TLA among treatments, and therefore, that analysis of the effect of TLA on fruit growth could be done on the basis of computed TLA rather than using the treatment labels “high,” “medium,” and “low” as these were in fact more or less arbitrary labels employed before the accurate computation of TLA.

### Impact of Treatments on Light Response of Leaves

**Figure 3:**
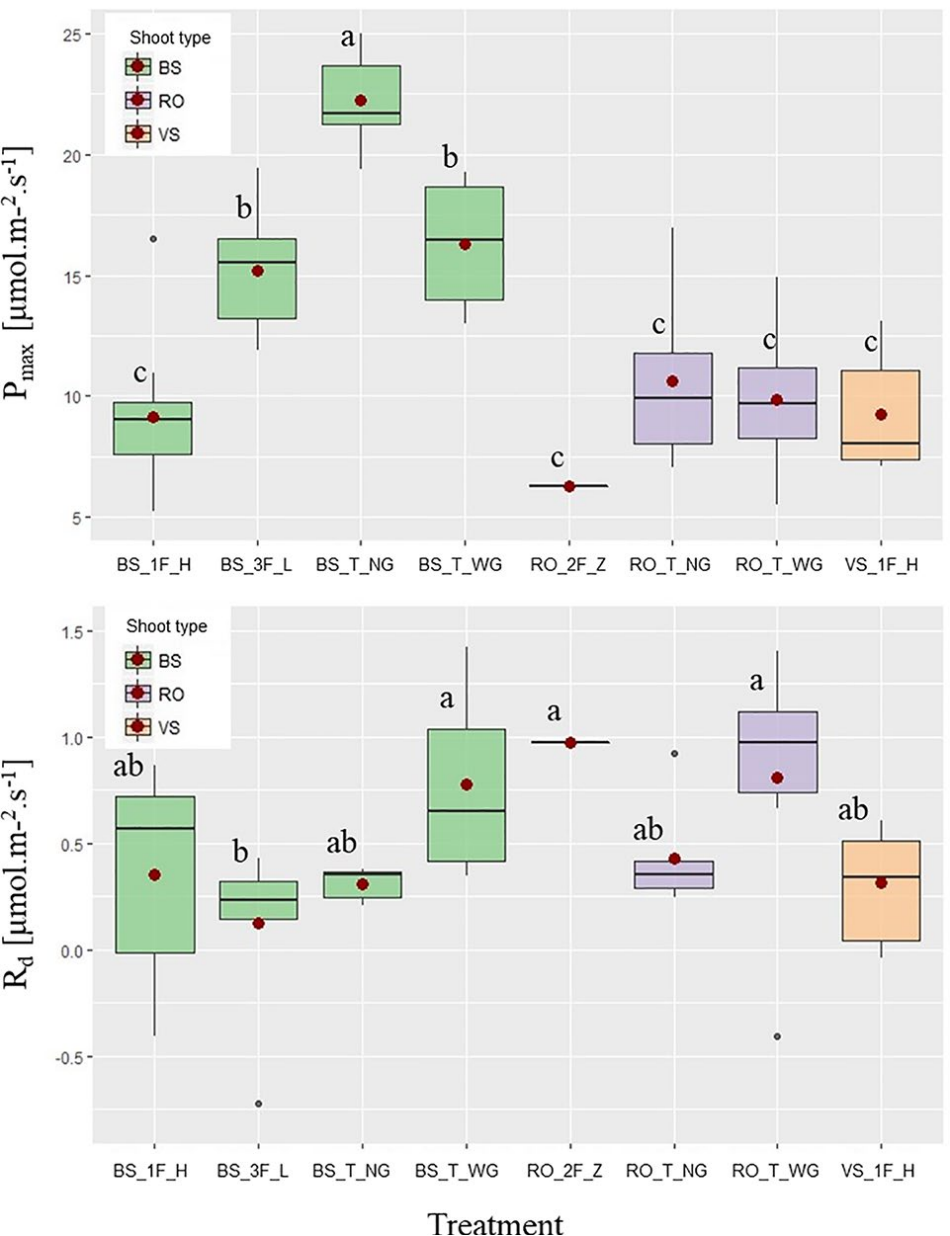
Boxplots of PAR saturated maximum photosynthesis rate [*P*_max_ (μmol·m^−2^·s^−1^)] and dark respiration rate [*R_d_* (μmol CO_2_·m^−2^·s^−1^)] as a function of different pruning and fruit removal treatments on “Fuji” branches: bourse shoot (BS) leaves on branches with one axillary fruit and high leaf area (“1_F_H”), with three axillary fruits and low leaf area (‘3F_L’), with one fruit on the terminal spur without girdling (“T_NG”) and with one fruit on the terminal spur with a girdling at the base of the spur (‘1F_T_WG’). Vegetative shoot (VS) leaves on branches with one axillary fruit and high leaf area (“1F_H”); and rosette (RO) leaves on spurs in terminal position without girdling (“T_NG”) and with girdling (“T_WG”), as well as with two axillary fruits and almost zero leaf area (“2F_Z”). Mean values for each group of leaves (•). Groups of leaves with the same letter are considered to be non-significantly different according to one-way ANOVA results (*P* < 0.05).

ANOVA and/or Kruskal–Wallis showed that the parameters α and θ of the Marshall and Biscoe model were not different among groups of leaves (data not shown), in contrast to *P*_max_ and *R_d_* (**Figure 3**). For *P*_max_, three different groups were distinguished; a high *P*_max_ in terminal bourse shoot leaves (“BS_T_NG”), an intermediate *P*_max_ in leaves of “BS_T_WG” and “BS_3F_L,” and finally, a low *P*_max_ in all other leaves. As for the dark respiration rate *R_d_* relatively high values were found in rosette leaves on totally defoliated branches with two fruits (“RO_2F_Z”), terminal rosette leaves (“RO_T_WG”), and terminal bourse shoot leaves (“BS_T_WG”) (**Figure 3**); while a relatively low *R_d_* was found in only one treatment (three fruits and “low” leaf area, “BS_3F_L”), with the remaining treatments showing intermediate values that did not differ from the two other groups (**Figure 3**).

### Impact of Treatments on Fruit Growth

In “Fuji,” initial fruit diameter measured at the onset of the experiments showed no difference with respect to their “rank” (topological distance of fruit to the sources: first, second, or third fruit) in branches with two and three axillary fruits, respectively (ANOVA, *P* = 0.194). This was also the case in branches with two axillary fruits in “Ariane” (ANOVA, *P* = 0.534). Moreover, initial fruit diameters did not show any differences among treatments in “Fuji” and “Ariane,” respectively. Therefore, it was considered that, at the onset of the experiment, axillary fruits were equivalent among their ranks on the branch and among treatments in both genotypes (data not shown). However, initial fruit diameter was different among genotypes (Kruskal–Wallis test, *P* < 2.2 × 10^−16^); therefore, in the following, fruit growth and response of fruits to treatments will be treated separately for each genotype.

Distance of a sink (fruit) to the source did not have any impact on either the asymptote (*D*_max_) or the slope (*s*) of the von Bertalanffy model used to describe fruit growth in diameter. Moreover, the distance of a fruit to the next source leaf had no effect, neither on fruit fresh and dry weight at harvest, nor on fruit dry matter content at harvest (**Table 2**).

**Table 2:** Results of ANOVA tests of the impact of the rank of the sink and leaf area available per fruit, on model parameters *D*_max_ and *s*, fruit fresh weight at harvest, fruit dry weight at harvest and fruit dry matter content at harvest.

Genotype	Variable	Sink ranking		Leaf area		Sink Ranking x Leaf area	
		2 fruits	3 fruits	2 fruits	3 fruits	2 fruits	3 fruits
Fuji	parameter *Dmax*	ns	ns	***	ns	ns	ns
	parameter *s*	ns	ns	ns	ns	ns	ns
	Fresh weight	ns	ns	***	ns	ns	ns
	Dry weight	ns	ns	***	ns	ns	ns
	Dry matter content	ns	ns	***	ns	ns	ns
Ariane	parameter *Dmax*	ns	–	***	–	*	–
	parameter *s*	ns	–	ns	–	**	–
	Fresh weight	ns	–	***	–	*	–
	Dry weight	ns	–	***	–	*	–
	Dry matter content	ns	–	***	–	ns	–

D_max_ and s were parametrized for each fruit using the model by von Bertalanffy (equation 6). Two fruits, 3 fruits: branches bearing two and three fruits, respectively, with no fruit drop until harvest (n = 21 and 15 on branches with 2 fruits, of ‘Fuji’ and ‘Ariane’, respectively and n = 5 on branches with three fruits, of Fuji). *, ** and *** for P values < 0.05, 0.01, and 0.005.

**Figure 4:**
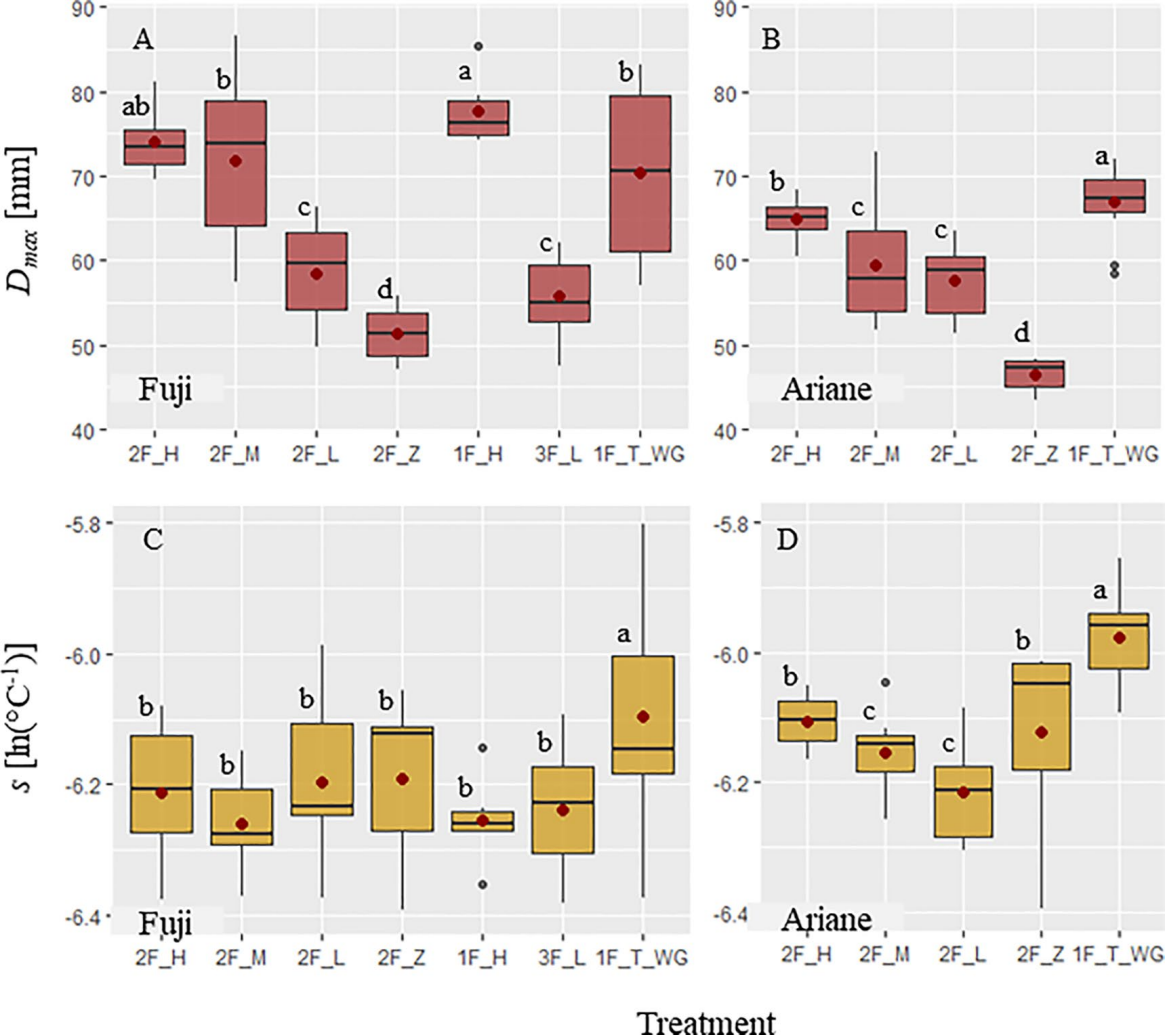
Boxplots of **(A**, **B)** maximum fruit diameter *D*_max_ [mm] and **(C**, **D)** parameter *s* [ln(°C^-1^)] parameterized for each fruit using the von Bertalanffy model (equation 6) as a function of different pruning and fruit removal treatments on ‘Fuji’ (FU) and ‘Ariane’ (AR) branches, respectively: For treatment labels see Materials and Methods. Mean values for each treatment (•). Treatments with the same letter are considered to be non-significantly different according to one-way ANOVA and/or Kruskal–Wallis results (*P* < 0.05).

Growth in fruit diameter, from the start of the experiment to harvest, showed distinct patterns among treatments for both genotypes, expressed in terms of *D*_max_ and *s* (**Figure 4**). In “Fuji,” the highest values of *D_max_* were observed in axillary fruits of treatments with “high” and “medium” leaf area (“1F_H,” “2F_H,” and “2F_M”) and in terminal fruits (“1F_T_WG”), whereas the lowest values were observed in axillary fruits on branches with no leaves (“2F_Z”). Treatments with axillary fruits with “high” leaf area showed the highest mean *D_max_* (**Figure 4A**). In “Ariane,” terminal fruits had the highest mean of *D_max_*, while axillary fruits on branches with no leaves exhibited the lowest *D_max_* (**Figure 4B**). These results indicate that in axillary fruits, *D_max_* was increasing with available leaf area. Parameter *s* is linked to the slope of the curve, which means that fruit growth rate increased with increasing *s*. In both genotypes, *s* was highest in terminal fruits. However, *s* was not different among treatments for axillary fruits in “Fuji” (**Figure 4C**). In addition, in “Ariane,” *s* was higher in axillary fruits with “high” and “zero” treatments compared to those with “medium” and “low” treatments (**Figure 4D**). However, these results might depend on the variability of leaf area available per fruit in each treatment and the fact that branches did not always exhibit differences in leaf area as a function of treatments.

### Impact of Leaf Area Available Per Fruit on Fruit Growth

**Figure 5:**
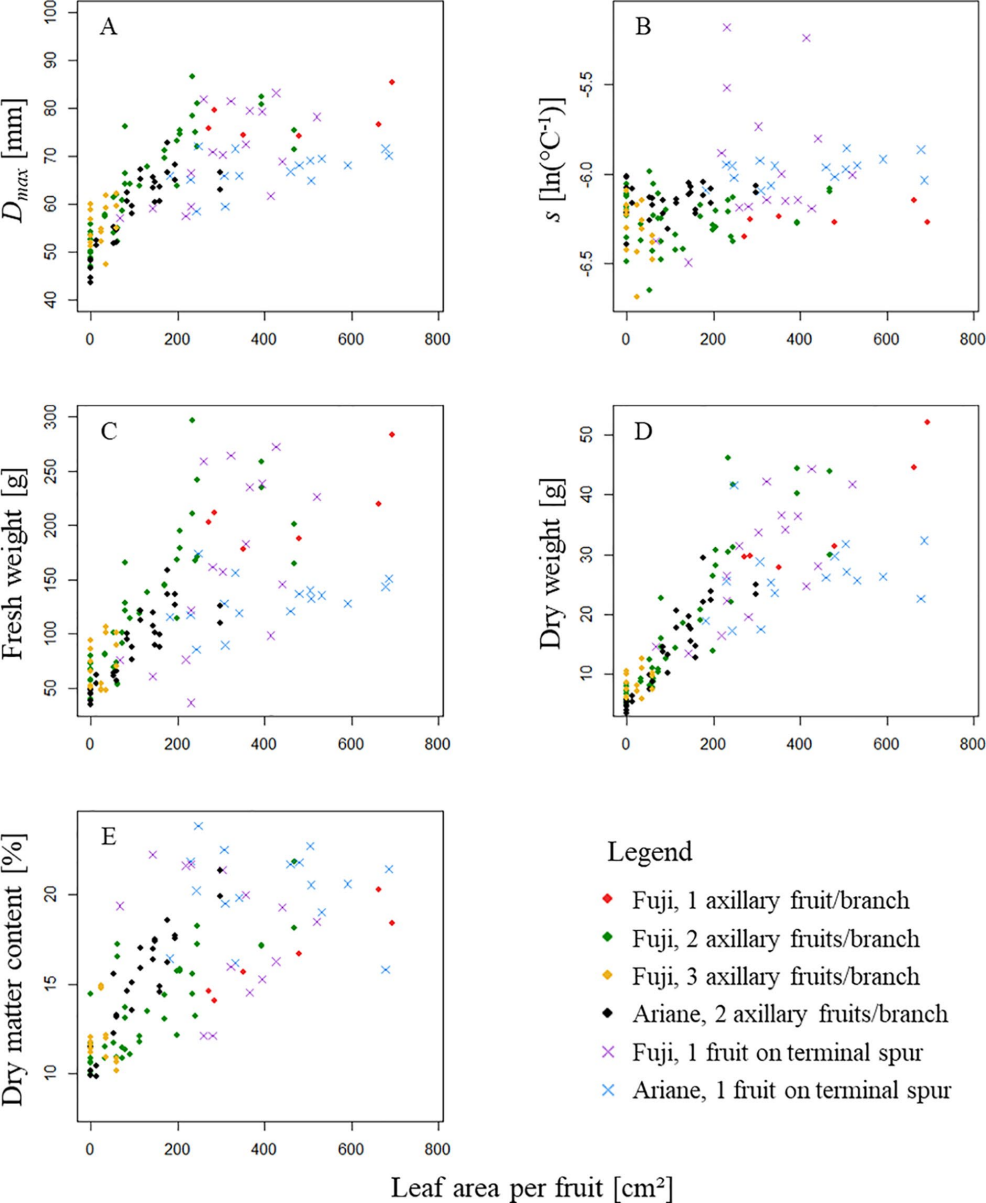
Distribution of **(A)** maximum fruit diameter *D_max_* [mm], **(B)** the parameter *s* [ln(°C^-1^)] parametrized for each fruit using Von Bertalanffy model (equation 6), **(C)** fruit fresh weight at harvest [g], **(D)** fruit dry weight at harvest [g] and **(E)** fruit dry matter content at harvest [%] as a function of leaf area available per fruit [cm²]. In the legend, “1 axillary fruit/branch” refers to one axillary fruit with ‘high’ leaf area; “2 axillary fruits/branch” refers to two axillary fruit with ‘high’, ‘medium’, ‘low’ and almost ‘zero’ leaf area (“2F_H,” “2F_M,” “2F_L,” “2F_Z”); “3 axillary fruits/branch” refers to three axillary fruits with “low” leaf area treatment (“3F_L”); and “1 fruit on terminal spur” refers to treatment with one fruit on girdled terminal spur.

**Figure 5** shows the distribution of five parameters describing fruit response to treatments as a function of the leaf area available per fruit (*ALA*). These parameters were: *D*_max_ and *s* established for fruit growth curves, fresh weight of the fruit at harvest (*FW*, g), dry weight of the fruit at harvest (*DW*, g) and dry matter content of the fruit at harvest (*DMC*, -). The figure shows equivalent patterns for axillary fruits, which seem unrelated to treatments and genotypes. However, fruits on terminal spurs of both genotypes showed quite different patterns. Moreover, treatments with three axillary fruits and “low” leaf area (“3F_L”) were very similar in terms of leaf area available per fruit, and in fact, the points were too close to each other to show a coherent pattern. Tests made on axillary fruits to check if *ALA* had an impact on the aforementioned five parameters for each group of axillary fruits [“Fuji,” one axillary fruit/branch; (ii) “Fuji,” two axillary fruits per branch; (iii) “Fuji,” three axillary fruits per branch; and (iv) “Ariane,” two axillary fruits per branch] consistently showed a significant impact of *ALA* on *D_max_*, *FW*, *DW*, and *DMC* except in “Fuji,” three axillary fruits per branch (“3F_L” treatment) (data not shown). However, as indicated above, leaf area in this treatment was too low to be correctly analyzed, and *ALA* did not have a significant impact on *s* in all groups of axillary fruits.

Moreover, treatments with axillary fruits showed a logistic pattern for maximum fruit diameter *D_max_* as a response to *ALA* (**Figure 5A**). When considering each group of axillary fruits separately, all *FW*, *DW*, and *DMC* as a function of *ALA* seemed to follow a linear pattern. In particular, if we assume that i) sugars provided by reserve remobilization were negligible, ii) rosette leaves near the fruit were equal in their sugar supply, and iii) the girdling provided isolation of the branch from the rest of the tree, dry weight of the fruit should indicate the amount of sugars supplied by leaves from the beginning of the experiment until harvest. As the pattern was linear for each group of axillary fruits [i.e., i) “Fuji,” one axillary fruit/branch, ii) “Fuji,” two axillary fruits per branch, iii) “Fuji,” three axillary fruits per branch, and iv) “Ariane,” two axillary fruits per branch], it is assumed that for each group, the increase in leaf area per fruit was proportional to fruit increase in diameter (**Figure 5B**); however, the figure suggested that the slope varied with the number of fruits per branch but not with genotype when considering the same fruit load (two axillary fruits per branch). Therefore, a linear regression model was used to describe fruit dry weight at harvest (equation 7).

(7) $$DW_{H}(ALA)=DW0_{G,n}+p_{G,n}\cdot ALA$$

*DW_H_* is fruit dry weight at harvest; *ALA* is the leaf area available per fruit; *DW0_G,n_* is “minimal” fruit dry weight at harvest when all leaves were removed except the rosette leaves of the fruit itself, and *p_G,n_* is the slope of the curve for branches bearing *n* axillary fruits of a genotype *G*.

Regression models *M_FU,1_*, *M_FU,2_*, and *M_AR,2_* established for dry weight at harvest *DW_H_* in “Fuji” (1 and 2 axillary fruit/branch), and “Ariane” (two axillary fruits per branch) as a function of leaf area available per fruit (*ALA*) showed coefficients of determination that indicate that in all three cases the increase in fruit dry weight at harvest was proportional to the increase in leaf area available per fruit. Moreover, the slopes of *M_FU,2_* and *M_AR,2_* were quite similar, whereas *M_FU,1_* had a smaller slope coefficient (**Table 3**). This indicates that treatments with two axillary fruits per branch exhibited the same rate of increase in *DW_H_* as a function of *ALA*, while in the treatment with one axillary fruit this rate was lower. Moreover, the ratio between *p_FU,2_* and *p_FU,1_* of the models *M_FU,2_*, *M_FU,1_*, respectively, was equal to 1.71, which suggests that in “Fuji,” the same leaf area per fruit in branches bearing two fruits provided 71% more dry matter to the fruit than in branches bearing only a single fruit, and this for a large range of leaf areas per branch.

**Table 3:** Output of regression models *M*_FU,1_, *M*_FU,2_, and *M*_AR,2_ for dry weight at harvest (DW_H_) in “Fuji,” 1 axillary fruit/branch; “Fuji,” 2 axillary fruits per branch; and ‘Ariane’, 2 axillary fruits per branch, respectively, as a function of leaf area available per fruit (*ALA*) (see Equation 7).

Model	DW0_G,n_			*p*_G,n_				
	Value	*P*-value		Value	*P*-value		R²	adj_R²
M_FU,1_	13.38	0.06018	ns	0.049	0.00957	**	0.84	0.81
M_FU,2_	7.51	0.000529	***	0.083	7.93e-10	***	0.72	0.71
M_AR,2_	6.39	0.000317	***	0.074	2.74e-07	***	0.71	0.69

ns, non-significant. *, **, and *** for P value < 0.05, 0.01, and 0.005.

**Figure 6:**
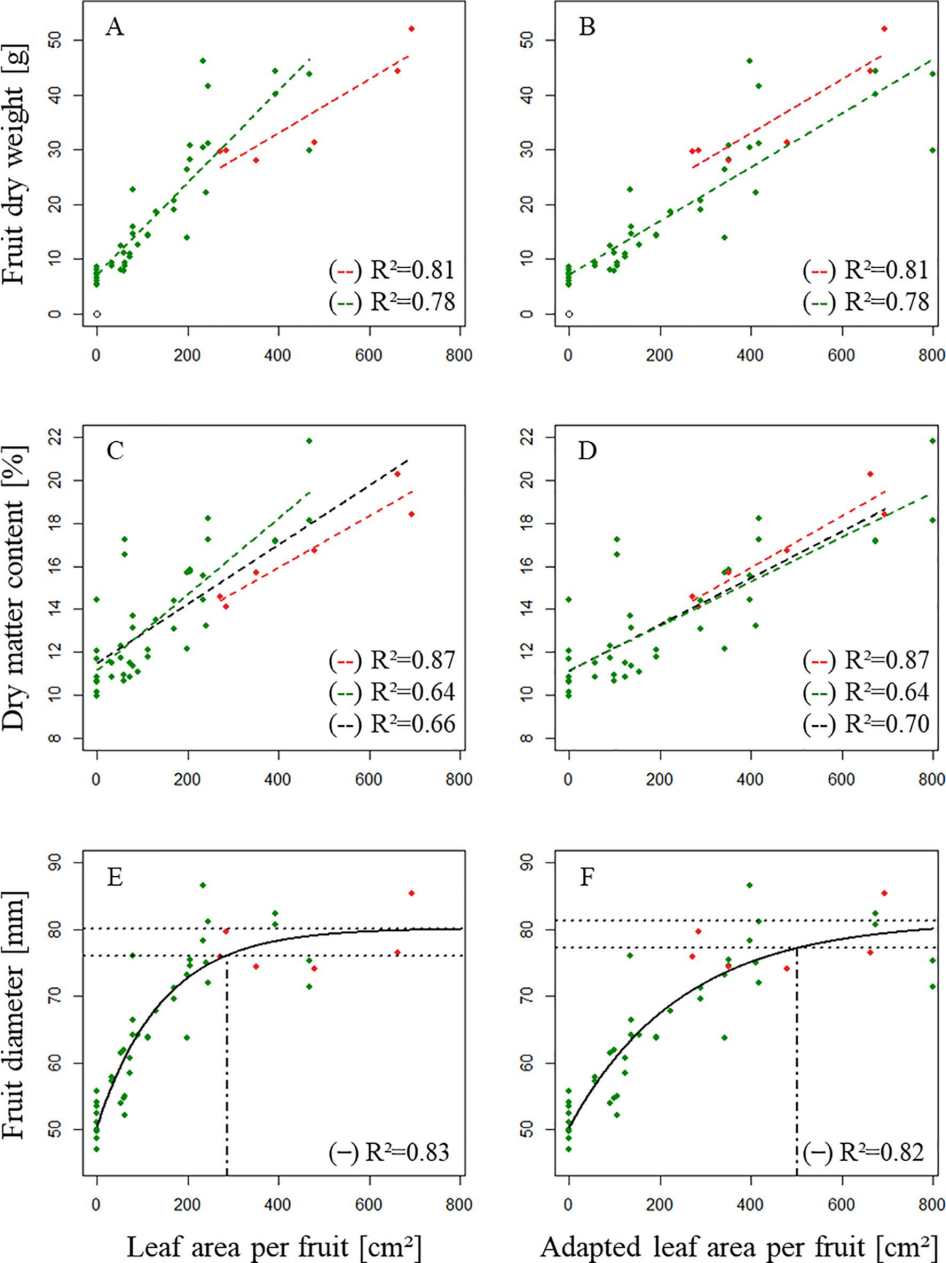
Distribution of **(A**, **B)** fruit dry weight at harvest [g], **(C**, **D)** dry matter content of the fruit at harvest [%] and **(E**,** F)** the highest diameter of the fruit observed during its development *D*_max_ [mm] as a function of leaf area available per fruit (**A, C, E**; [cm^2^]) and adapted leaf area available per fruit **[B**, **D**, **F**; (cm^2^)]. (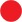): One fruit/branch; (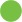): Two fruits/branch. In E, F, the two dotted horizontal lines delimit the upper 5% of the model output (equation 9) and the vertical dot dash line corresponds to the minimum leaf area necessary for potential growth.

Therefore, to take into account the upregulation of the assimilation rate of a given leaf area compared to the one obtained when only a single sink was present on the branch, we introduced a coefficient λ*_FU,n_* as well as a variable “adapted available leaf area” (*AdALA*) (Equation 8):

(8a) $$AdALA=\lambda_{FU,n}\cdot ALA$$

(8b) $$\lambda_{FU,1}=\{\begin{array}{cc} 1 & if\;N_{fr}=1\\ \frac{p_{FU,2}}{p_{FU,1}} & if\;N_{fr}=2 \end{array}$$

where *N_fr_* is the number of fruits per branch. Afterward, we modeled fruit dry weight at harvest, dry matter content at harvest, and parameter *D*_max_ as a function of *ALA* [**Figure 6 (A, C, E)**] and as a function of *AdALA* [**Figure 6 (B, D, F)**], respectively for axillary fruits of “Fuji.”

A regression model using *AdALA* as input allowed a better adjustment of simulating dry matter content when combining treatments of branches bearing both one and two fruits (*R*² = 0.70, **Figure 6D**) compared to a regression model using *ALA* as input (*R*² = 0.66, **Figure 6C**). *D*_max_ was modeled as a function of leaf area per fruit using the *nls* function in R based on a negative exponential model (Equation 9).

(9) $$D_{max}(LA)={∆}_{pot}(1-e^{-k,LA})+D_{s}$$

*D*_max_ (mm) is estimated as a function of *LA* (cm²) (*LA* = *ALA* in **Figure 6E** and *LA* = *AdALA* in **Figure 6F**). Δ_pot_ (mm) is the difference observed between the asymptote of model 9 (which corresponds to potential diameter at harvest) and minimal diameter at harvest [*D_s_* (mm)]. Parameter *k* (cm^-1^) describes the curvature of the model. Initial parameters for the *nls* function in *R* were fixed to Δ_pot_ = 20, *k* = 0.004, and *D_s_* = 55.

Fits of *D*_max_ using both *AdALA* and *ALA* (**Figures 6E, F**) showed a good fit.

In this study, it has been decided to accept the upper 5% of the output of the model as potential fruit diameter. Leaf area necessary for potential growth was therefore estimated to be 286 cm² when using *ALA* and to be 502 cm² when using *AdALA*.

Models also showed that *DW* at harvest differed with fruit number per branch; however, this result cannot be confirmed, as we did not test the case of having one fruit with no or “low” leaf area on the branch. Models showed that dry matter content was similar among branches, independent of fruit number (1 or 2), even when the branch did not bear any leaves (except for the rosette leaves). This suggests that fruit dry matter content in “Fuji” was not affected by treatments.

## Discussion

The present work aimed at the understanding of the dynamic interactions between sources and sinks in apple at the scale of the (first-order) fruit-bearing branch.

In the present study, TLA per shoot and per leaf was not measured but predicted using an allometric model (Baïram et al., 2017). Using this model, we computed individual and per-shoot leaf areas to quantify *a posteriori* the actual TLA of the experimental branches that prior to this, had just been labeled as “High,” “Medium,” or “Low.”

The first aim of this study was to investigate the effect of source–sink distance on fruit growth. Our results indicate no significant differences among fruits belonging to different ranks, and this independent of source availability. These results, therefore, seem to confirm those obtained by Hansen (1969), who showed that the distribution of carbon between sources and sinks could not be explained exclusively by source–sink distance. What is more, these results suggest that carbon allocation takes place in a similar and homogeneous manner among all fruits on the same branch. Indeed, such a result is in contradiction with those obtained by Hansen (1969), who, though not demonstrating a distance effect for carbon allocation, nevertheless provided some evidence for an unequal distribution of carbon among the different fruits, which he attributed to the preferred vascular connections between certain leaves and fruits at the scale of the fruiting branch. Still, this hypothesis, which was taken up by other authors (Watson and Casper, 1984; Dengler, 2006) and attributed to the phyllotaxy of the plant (Barlow, 1979; Orians et al., 2005; Dengler, 2006) remains controversial since the differences might also be explained by differences in initial sink strength among fruits of the branch, contrary to our study in which the initial size of fruits, and thus *a priori* their sink strengths, were statistically tested to be equal. It would therefore be interesting, in the frame of a follow-up study, to simultaneously test the effect of initial fruit size and of source–sink distance to know which one of these two factors is the most determinate for carbon allocation at the level of the fruit-bearing branch in apple. Although in the present study we did not notice an effect of source–sink distance on fruit growth, we nevertheless identified an effect of the position of the fruit on the branch: Indeed, we observed a clear effect of apical dominance when comparing terminal with lateral spurs. This effect was visible for all fruit growth-related traits.

Our second objective was the investigation of the effect of the source/sink ratio on fruit growth and leaf photosynthetic rate. We found evidence that not only the leaf area available per fruit (as usually assumed) is determinant for dry matter accumulation in fruits but also the number of growing fruits (sinks). This was surprising as it contradicted the literature and common orchard practice. Effectively, our results tend to show that a modification of the source/sink ratio is compensated by an alteration of the photosynthetic rate of leaves, with stronger and weaker values obtained for weaker and stronger ratios, respectively. Of course, this interpretation must be taken with great caution and not be generalized as in our experiments we covered a rather limited range of source–sink ratios, especially neglecting very low ratios (with a high number of fruits). Also, the time we took in between measurements at a given light level (30 s) might have been too short: photosynthesis rate might not have been at steady-state after this time, and in addition, we might have experienced a lowered quantum yield (slope between PAR and photosynthesis in the 0–200 µmol range) because of effects of photoinhibition. On the other hand, the observations made in our study have already been made by other authors (Palmer, 1992; Blanke, 1997; Wünsche et al., 2005) and could be explained by a modulation of the foliar concentration of starch, which latter is known to have an inhibitory effect on stomatal conductance (Roden and Ball, 1996), as a function of carbon demand. Again, this result was only quantified for branches with one and two fruits, but it is an interesting finding and puts into a wholly different light the definition of potential growth or rather, the method to obtain it, which is usually by reducing the number of sinks, thereby obtaining a high (saturating) source–sink ratio. Moreover, our results seem to suggest that two growing sinks together will upregulate the photosynthetic rate more strongly than one growing sink on its own, and this with the same leaf area per fruit. However, to ascertain this claim, we should have conducted measurements on experimental branches that combined two fruit with variable leaf area (H, M, L). As for the likely mechanisms involved in this upregulation, we can only speculate: first, perhaps two sinks achieve a more rapid depletion of stored starch from intermediate stores (leaves), thereby lifting product inhibition of photosynthesis (Roden and Ball, 1996); second, as suggested by Sané et al. (2012) two fruits could induce an increase in vascular area that could facilitate carbon transport by reducing flow resistance; or third, competition between the two fruits could induce an increase in their sink activity, which in turn could induce an increase in their sink strength and thus their ability to attract carbon. On the other hand, we cannot generalize our findings to normal conditions. In our experiments, we worked with rather small, girdled branches, which represent closed systems with respect to carbon as well as slightly suboptimal systems with respect to water flow and transpiration because of the xylem that is exposed to the air at the girdling zone. What is more, at the base of a girdled branch, upstream of the girdling zone, assimilates destined for export to the tree trunk and roots will accumulate, which usually leads to a downregulation of photosynthesis rate (Poirier-Pocovi et al., 2018). Our results nevertheless suggest that to obtain a satisfactory source sink-ratio (i.e., one that guarantees near-potential fruit growth) at the scale of the fruit-bearing branch, one should take into consideration the number of fruits available for a given leaf area per fruit. One should then evaluate the source–sink ratio using an “effective” leaf area rather than the apparent leaf area, where the former includes both a quantification of photosynthetic efficiency and area available for photosynthesis.

## Conclusion and Perspectives

In addition to improving the understanding of the mechanisms surrounding apple source–sink relationships, the present study also provides a set of parameters and initial architectures for an FSPM of the apple branch that we are currently developing (Buck-Sorlin et al, in preparation). This model has been conceived to integrate the different results and insights gathered during studies like the present one, and to help interpret the findings by simulating source–sink dynamics under different assumptions and hypotheses with respect to the rigidity or flexibility of model parameters such as fixed versus up- or down-regulated carbon assimilation rate. It is essential for such a model to have an accurate characterization of sources and sinks, more specifically of leaf area and fruit growth dynamics, to describe the initial branch architecture and the sink behavior as accurately as possible, with the ultimate aim to derive hidden parameters such as transport coefficients for carbon and water. Allometric models such as the one we created to predict shoot and individual leaf area (Baïram et al., 2017) are very helpful tools to faithfully predict more complex and difficult to measure traits from variables that are easy to score (leaf number per shoot). The faithful description of 3D structure, with all shoot types and leaves positioned correctly in space, is a prerequisite for the accurate prediction of light interception and thus photosynthesis. First test runs, in which the model was applied to one of the measured branches, were very promising, yielding an error of less than 1% (Gerhard Buck-Sorlin, unpublished results). The complete model will be the subject of a follow-up paper.

## Author Contributions

All authors conceived the experiments. EB, CM, and GB-S conducted the experimental work. EB carried out the statistical data analysis and modelling. EB, MD, and GB-S interpreted the results and contributed to all parts of the manuscript. All authors approved of the final version.

## Funding

Financial aid was provided by a strategic grant by the Région Pays de la Loire, grant 2012–12016.

## Conflict of Interest Statement

The authors declare that the research was conducted in the absence of any commercial or financial relationships that could be construed as a potential conflict of interest.
